# WoundMx: Multiplexed Detection of Wound Infection Biomarkers with a Multimodal Sensor System based on Laser-Induced Graphene

**DOI:** 10.21203/rs.3.rs-7274589/v1

**Published:** 2025-08-20

**Authors:** Heshmat Asgharian, Vinay Kammarchedu, Scott J. Gu, Aida Ebrahimi

**Affiliations:** 1 Department of Electrical Engineering, The Pennsylvania State University, University Park, PA, 16802, United States; 2 Materials Research Institute, The Pennsylvania State University, University Park, PA, 16802, United States; 3 Center for Atomically Thin Multifunctional Coatings, The Pennsylvania State University, University Park, PA, 16802, United States; 4 Department of Biomedical Engineering, The Pennsylvania State University, University Park, PA, 16802, United States; 5 Center for Neural Engineering, The Pennsylvania State University, University Park, PA, 16802, United States

**Keywords:** Laser-Induced Graphene, Wound Monitoring, Multiplexed Detection, Multimodal Sensing

## Abstract

Real-time, multiplexed monitoring of wound infection biomarkers is essential for early detection of infection and inflammation, as well as for evaluating wound healing progression. However, existing biosensing technologies lack the sensitivity, specificity, and integration needed to meet these clinical demands. To address current limitations in wound monitoring, we developed a portable and multimodal sensor system capable of simultaneously detecting uric acid (UA), phenazine-1-carboxylic acid (PCA), interleukin-6 (IL-6), and pH. The device integrates laser-induced graphene (LIG) electrodes with differential pulse voltammetry for UA and PCA sensing, open-circuit potential sensing using polyaniline-modified LIG for pH, and an extended-gate field-effect transistor-based component for IL-6 detection. We validated the performance of the sensors in phosphate-buffered saline and a wound simulating medium, demonstrating accurate, multiplexed detection across clinically relevant ranges. Furthermore, *in-vitro* experiments with an agar-based wound model highlighted the system’s promise for future application in wound monitoring. A custom-designed wireless analyzer was integrated to enable real-time, multiplexed data acquisition at the point-of-care. This minimally invasive platform offers a comprehensive solution for continuous wound assessment, with potential applications in infection detection, inflammatory disease monitoring, and next-generation smart wound dressings.

## Graphical Abstract

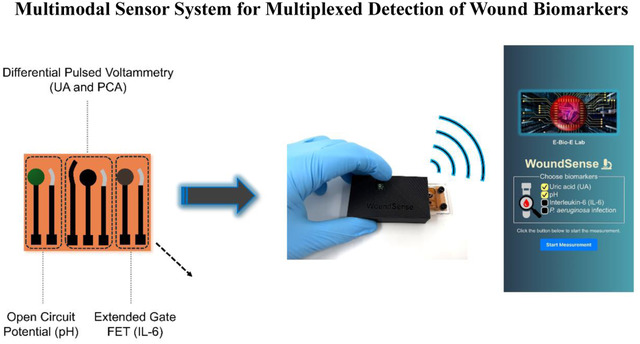

A wireless multimodal biosensor based on laser-induced graphene enables real-time, multiplexed detection of uric acid, phenazine-1-carboxylic acid, interleukin-6, and pH for wound assessment. By integrating electrochemical and EGFET transduction modes, the platform achieves sensitive and reliable performance in wound-simulating media and agar-based tissue-mimicking models, supporting continuous monitoring of infection, inflammation, and healing progress.

## 1. Introduction

Chronic wounds, such as diabetic foot ulcers, and acute injuries including severe burns, represent a major challenge for the global healthcare system due to their high treatment costs and adverse impact on patient outcomes. In the United States alone, chronic wounds affect more than 6.5 million individuals and result in over $25 billion in annual healthcare expenditures.^1^ According to industry estimates, treatment of severe burn injuries can exceed $1 million per case.^2^ Delayed or inadequate diagnosis and treatment often lead to infection, with *Staphylococcus aureus* and *Pseudomonas aeruginosa* (*P. aeruginosa*) being the predominant pathogens—accounting for approximately 30 − 40% and 10 − 20% of infections, respectively.^3,4^ These wounds are especially vulnerable to polymicrobial infections, where synergistic interactions among multiple bacterial species further complicate treatment and elevate the risk of severe complications such as sepsis.^5,6^

Effective treatment of wounds relies on early and accurate diagnosis to enable timely intervention and improve patient outcomes. Leveraging advances in materials science and bioanalytical methods, biosensors have emerged as powerful tools for the sensitive and specific detection of a wide range of health conditions.^7–10^ In this context, the early and easy identification of various wound biomarkers is becoming increasingly acknowledged as essential for early diagnosis and treatment of wound infections.

For assessing wound status, monitoring a combination of metabolic, microbial, inflammatory, and physicochemical biomarkers enables a thorough understanding of patient health. For instance, in wound exudate, uric acid (UA) levels generally vary from 220 to 750 *μM* in healthy conditions. UA level below ca. 220 *μM* indicates infection by microbial pathogens as they convert UA into 5-hydroxyisourate using microbial uricase, which is not present in humans.^11^ On the other hand, an increase above 750 *μM* signifies tissue necrosis, as significant cell death leads to the release of adenosine triphosphate (ATP), which later breaks down into UA.^11^ In systemic conditions such as gout, the serum UA levels surpass 360 *μM*, resulting in supersaturation and the development of monosodium urate crystals.^12^

Among various microbial infections, *P. aeruginosa* is a notable pathogen, responsible for approximately 7–10% of hospital-acquired infections. It poses a particularly high risk to immunocompromised patients, individuals with cystic fibrosis, and those with chronic wounds, often leading to elevated mortality rates in these populations.^4,13^ Most environmental strains of *P. aeruginosa* have developed antibiotic resistance, which makes conventional antibiotic treatments ineffective, thus resulting in high mortality rates.^14^ *P. aeruginosa* produces redox-active chemicals called phenazines (mainly phenazine-1-carboxylic acid: PCA and pyocyanin: PYO) which serve as early indicators of infections.^15^ PCA is a direct biosynthetic precursor to PYO, with the enzymes PhzM (methyltransferase) and PhzS (monooxygenase) catalyzing its conversion. Monitoring PCA provides an intrinsic readout of PYO biosynthetic pathway activity.^16^ This pathway plays a key role in the virulence of *P. aeruginosa*. Besides, PYO impedes wound healing by inducing cell cycle arrest in a concentration-dependent way, within a range of 1 − 50 *μM*.^17^ Notably, PCA demonstrates greater stability than pyocyanin, exhibiting a half-life exceeding 10 days, compared to approximately 1 day for PYO.^18^

Cytokines, such as interleukin-6 (IL-6), are another important indicator of wound infection and healing. IL-6 plays a crucial role in both pro-inflammatory and anti-inflammatory processes.^19^ Its levels rise significantly during sepsis, cytokine storms, and chronic inflammation, contributing to approximately 11 million deaths globally each year.^20,21^ Thus, tracking IL-6 levels can serve as an early signal of severe infections and inflammatory disorders, allowing for prompt medical action.^22^ In healthy wound exudate, IL-6 levels are about $0.2\;\frac{ng}{ml},$ increasing to $\sim 3\;\frac{ng}{ml}$ in chronic wounds, whereas in sepsis, serum IL-6 levels often exceed $500\;\frac{pg}{ml}$.^23–25^

**Scheme 1.**
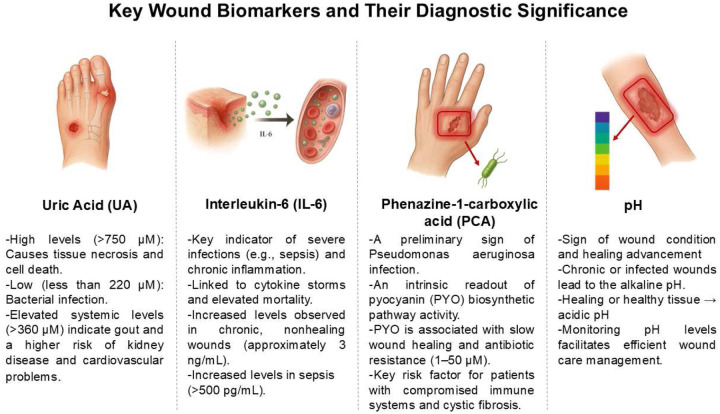
Schematic illustration showing different wound biomarkers. UA, IL-6, PCA, and pH serve as example key indicators of metabolic, microbial, inflammatory, and physicochemical state of wounds. Simultaneous analysis of these biomarkers provides a comprehensive view of infection status, inflammatory response, and healing progression, enabling the development of personalized wound management strategies. The schematics were created with the assistance of ChatGPT (OpenAI).

Monitoring the pH level in wounds is another indicator for evaluating wound condition and determining suitable treatment. The local pH of wounds serves as a well-recognized indicator of their status: chronic or infected wounds generally exhibit an alkaline pH (approximately 7–9), while healthy skin and healing wounds tend to be more acidic.^26^ These examples highlight the clinical significance of tracking UA, PCA, IL-6, and pH. This combination of biomarkers reflects metabolic state, pathogenic infection, systemic inflammation, and local tissue condition, as illustrated in Scheme 1.

Traditional tests for these biomarkers, though considered the gold standard in centralized laboratories, are not appropriate for point-of-care applications. For UA, colorimetric assays using enzymes^27,28^ or high-performance liquid chromatography (HPLC)^29–31^ on blood/urine/saliva samples are commonly used, while PCA and PYO detection may involve HPLC,^32,33^ spectrophotometry,^34,35^ proton nuclear magnetic resonance,^36,37^ or Fourier transform infrared spectroscopy.^38^ IL-6 is commonly quantified using enzyme-linked immunosorbent assays^39^ or other immunoassays like chemiluminescent,^40^ and pH is frequently assessed using pH strips or benchtop meters.^41^ Although established methods are reliable, they often have slow turnaround times, requiring extensive sample processing and analysis that can hinder clinical decision-making. Moreover, these methods are often incompatible and depend on costly, bulky laboratory equipment, necessitating centralized facilities and trained personnel. As a result, their accessibility is limited, particularly in resource-constrained or decentralized settings. Traditional techniques are also poorly suited for continuous, real-time monitoring—an increasingly vital capability for managing dynamic physiological conditions such as sepsis or tracking wound healing progression.^42–48^

To address technical and clinical needs, we developed a portable, wireless biosensing platform based on laser-induced graphene (LIG) for the multiplexed detection of UA, PCA, IL-6, and pH. This multimodal system integrates electrochemical and extended-gate field-effect transistor readouts to enable comprehensive wound monitoring. LIG offers several advantages over traditional electrode materials such as metals and carbon nanotubes, particularly for wearable, multi-analyte sensors.^49–53^ The laser patterning process enables rapid, mask-free fabrication of electrodes in customizable geometries on flexible substrates, providing a cost-effective and scalable approach.^54,55^ Furthermore, LIG’s porous carbon-based structure delivers high electrical conductivity and a large specific surface area, enhancing electron transfer and allowing abundant sites for functionalization—such as antibody immobilization—critical for achieving low detection limits. These attributes make LIG an excellent candidate for multifunctional electrochemical biodevices.^56–58^

The developed LIG-based sensors exhibit high performance across all targets. For UA, the sensor achieved a sensitivity of $19.16\;\frac{\mu A}{dec}$ and a limit of detection (LOD) of 64.14 *μM*, within a dynamic range of 100 − 500 *μM*. PCA was detected with a sensitivity of $7.35\;\frac{\mu A}{dec}$ and an LOD of 0.88 *μM*, across a range of 100 *nM* to 50 *μM* using differential pulse voltammetry. The polyaniline (PANi)-modified LIG sensors enabled pH sensing via open-circuit potential measurements, covering a pH range of 4–8 with a sensitivity of $-54.16\;\frac{mV}{pH}$ The extended-gate field-effect transistor configuration allowed IL-6 detection from $5\;\frac{pg}{ml}$ to $50\;\frac{ng}{ml},$ with a sensitivity of $7.6\;\frac{mV}{dec}$ and an LOD of $0.05\;\frac{pg}{ml}.$ To our knowledge, this is the first report of a fully LIG-based multimodal sensing system that combines electrochemical and EGFET modalities for the simultaneous detection of metabolic, microbial, inflammatory, and physicochemical wound biomarkers. Sensor performance was validated in both phosphate-buffered saline (PBS) and a wound-simulating medium (WSM), demonstrating stable, accurate detection under physiologically relevant conditions. To further simulate real-world application, an agar gel layer was applied over the electrode arrays to mimic the wound surface, confirming the sensors’ ability to detect biomarkers through a soft, tissue-like interface. The system was integrated with a custom-designed wireless analyzer for real-time, multiplexed data acquisition. The developed portable multimodal sensor system represents a promising platform for personalized healthcare, particularly for at-home wound monitoring and infection tracking. By enabling rapid, simultaneous detection of multiple clinically relevant biomarkers, it can improve clinical decision-making and support early intervention in wound care.

## 2. Results

To enable multiplexed detection of key wound biomarkers, we developed a multimodal electroanalytical chip that integrates differential pulse voltammetry (DPV), open-circuit potential (OCP) sensing, and extended-gate field-effect transistor (EGFET) measurements—each implemented using LIG electrodes. The design, operating principles, and integration of these sensing modalities are detailed in the following sections.

### 2.1 pH Sensing with Polyaniline (PANI)-LIG Electrodes

**Figure 1.**
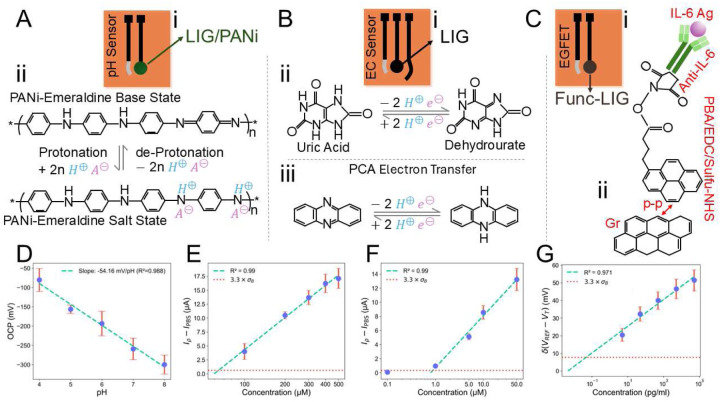
LIG-based multiplexed detection of pH, UA, PCA, and IL-6 using different sensing modalities. **(A)** Schematic illustration of the pH sensor with LIG/PANi as the working electrode (WE) and its sensing mechanism. **(B)** Schematic illustration of the electrochemical sensor for detecting UA and PCA. **(C)** Schematic illustration of the EGFET sensor with functionalized LIG as the gate and its sensing mechanism for detecting IL-6. **(D)** Calibration plot of OCP versus pH (n = 3) in McIlvaine buffer. The sensitivity (slope of the fitted line) is determined. **(E)** Calibration curves obtained using DPV after baseline subtraction for detection of UA (n = 9) and **(F)** PCA (n = 9) in 1× PBS. **(G)** Response to recombinant human IL-6 in 1× PBS, calculated based on the baseline-subtracted gate voltage at *I_DS_* = 150 *μA* (n = 9). The error bars show the standard errors. The dashed line represents linear fitting, and the dotted line represents the standard deviation of the blank solution (*σ_B_*).

The pH sensor measures the OCP of a PANi-modified LIG working electrode vs. a pseudo reference electrode (pRE) made with silver (Ag) paste, Figure 1 (A–i). The details of LIG fabrication process and characterization are provided in the Methods: LIG Fabrication, LIG Characterization and Figure S1. To make a PANi-LIG pH sensor, PANi is first electropolymerized on LIG, as described in Methods: pH Sensor Fabrication and Figures S2 (A) and (B). PANi, a conducting polymer, undergoes protonation and deprotonation that shifts its electrode potential based on the Nernst equation as pH changes. By measuring the OCP of a PANi-coated electrode against a reference electrode, we obtain a direct potentiometric readout of the local pH. This method serves as a compact, flexible solid-state alternative to traditional glass pH electrodes.^59,60^

As illustrated in Figure 1 (A–ii), the reversible protonation and deprotonation of the emeraldine state of PANi make the sensor sensitive to different pH levels. In acidic solutions, the emeraldine base structure of PANi is protonated with H^+^ and doped with an anion (A^−^), forming the emeraldine salt structure as a highly conductive form of PANi. On the other hand, the neutralizing effect of OH^−^ in alkaline solutions converts the conductive emeraldine salt form to the non-conductive emeraldine base form of PANi by deprotonation. Due to the change in resistance of PANi, the electrode’s OCP varies with a pH change.

To optimize the pH sensor, we conducted comprehensive comparisons of two common methods reported in the literature for preparing on-chip reference electrodes, namely Ag paste with and without polyvinyl butyral (PVB) modification^61–63^ (Methods: Preparation of the Modified Reference Electrode for pH Sensor), while also studying the effect of HCl surface cleaning. The detailed comparative results, sensitivity, linearity, and reproducibility, are provided in Supporting Information, Table S1, and Figure S3. In McIlvaine buffer solution (pH 4 − 8), the Ag paste reference electrode with HCl cleaning demonstrated a stable response, achieving a sensitivity of $-54.16\;\frac{mV}{pH}$ (*R*^2^ = 0.988, *n* = 3), which closely aligns with the theoretical Nernstian slope at room temperature $\left(\sim 59\;\frac{mV}{pH}\right)$.^64^ Figure 1(D) shows the variation of OCP as a function of pH during both ascending and descending pH cycles. Additionally, to demonstrate the critical role of PANi for sensing pH, we also tested bare LIG, see Figure S2 (C), showing a poor response and lack of reversibility compared to PANi-based LIG in Figure S2 (D).

### 2.2 Electrochemical Sensing of Uric Acid and Phenazine-1-Carboxylic Acid

Figure 1 (B) shows the electrode configuration and the reactions involved in the oxidation of UA and PCA. A pair of reversible peaks is associated with a two-electron oxidation of UA to dehydrourate, Figure 1 (B–i). Oxidation mechanism of PCA is also shown in Figure 1 (B–ii). For simultaneous detection of UA and PCA, we used DPV and developed calibration curves based on the signals at oxidation peaks between 0.3 to 0.4 V (for UA) and between −0.2 to −0.3 V (for PCA) (Figure S4). We first tested the sensors, built using 1-pass LIG, in 1× PBS. As shown in Figure 1(E), the sensor exhibited a sensitivity of $19.16\;\frac{\mu A}{dec}$ and a LOD of 64.14 *μM* for UA, with *R*^2^ = 0.99. Similarly, Figure 1 (F) demonstrates a sensitivity of $7.35\;\frac{\mu A}{dec}$ and an LOD of 0.88 *μM* for PCA, with *R*^2^= 0.99. We also evaluated the performance of sensors fabricated using 2-pass LIG for detecting UA and PCA in 1× PBS (Figure S5 (A) and (B)). Although these sensors showed slightly increased sensitivity (14.15% higher for UA, 11.16% higher for PCA), the chosen 1-pass LIG sensors provided lower limits of detection (21.48% lower for UA, 13.73% lower for PCA), allowing detection at lower analyte concentrations, which is advantageous for sensitive measurements.

Sensors were also tested in acidic pH (pH 5 and 6), corresponding to healthy or healing wound environments, as opposed to pH 7.4 (in 1× PBS) which is typically associated with chronic or infected wound conditions (Figure S6 and Table S2). The sensor performance was significantly affected by pH, showing increased sensitivity and altered LOD values for both UA and PCA under acidic conditions. Specifically, at pH 5, the sensor exhibited sensitivities of $37.14\;\frac{\mu A}{dec}$ for UA and $37.09\;\frac{\mu A}{dec}$ for PCA, whereas at pH 6, sensitivities were $19.45\;\frac{\mu A}{dec}$ for UA and $39.08\;\frac{\mu A}{dec}$ for PCA. Compared to pH 7.4, these results highlight the importance of performing pH-specific calibration when monitoring wound biomarkers. Such calibration ensures that changes in biomarker concentrations are accurately interpreted within the appropriate physiological context, ultimately facilitating precise and effective wound management.

### 2.3 EGEFET Sensors for Detection of IL-6

EGFET-based sensors offer several advantages over conventional FET platforms for biosensing applications. In EGFETs, the sensing electrode—referred to as the extended gate—is physically decoupled from the transistor. This separation enables direct exposure of the functionalized gate electrode (e.g., with IL-6 antibodies) to the sample solution, while keeping the transistor isolated from the chemical environment, thereby enhancing device stability and longevity.^65–70^

The sensing part of the LIG-EGFET developed in this study is schematically shown in Figure 1 (C-i,ii). The details of the EGFET device and functionalization of the extended gate are provided in the Methods: EGFET Configuration and Functionalization for IL-6 Detection. Example transfer characteristic of EGFET (I_DS_–V_REF_; V_REF_ = gate voltage) with different concentrations of IL-6 in 1× PBS is shown in Figure S7 (A), showing the increasing shift in the FET threshold voltage by increasing IL-6 concentration. Sensor sensitivity is calculated as $S_{v}=\frac{dv_{\text{REF}}}{d{\;\text{log}}_{10}(\text{Conc})}|I_{\text{DS}}=\text{const}$. and shown in Figure 1 (G), with *S_v_* of $7.6\;\frac{\text{mV}}{\text{dec}}$ rand LOD of $0.05\;\frac{pg}{ml}$ in our study. Additionally, the developed EGFET sensors show negligible hysteresis of 0.2 *mV* in 1× PBS across repeated measurements with IL-6 concentration of $500\;\frac{pg}{ml},$ as shown in Figure S8 (A)—such low hysteresis results from using a commercial MOSFET, which is important for a stable readout.

The EGFET sensors were also tested at other pH levels (Figure S9 and Table S3). Across all tested pH values (5, 6, 7, and 7.4), the LIG-based EGFET showed a consistent increase in threshold voltage with higher IL-6 concentrations. At lower pH (isoelectric point of IL-6 is 6.96^71^), the positively charged IL-6 molecules create a positive surface potential on the extended gate, which requires a higher reference voltage (*V_REF_*) to reach the same MOSFET gate bias and turn on the transistor channel. At near-neutral and slightly alkaline pH, even with minimal protein net charge, the adsorption of IL-6 forms interfacial dipoles that similarly alter the gate potential and shift the threshold voltage higher.^66,72^ This consistent rightward shift in the transfer curve supports a sensing mechanism based on both protein charge and dipole-related surface potential effects.

### 2.4 *In-Vitro* Detection of Wound Biomarkers in Wound-mimicking Environments

**Figure 2.**
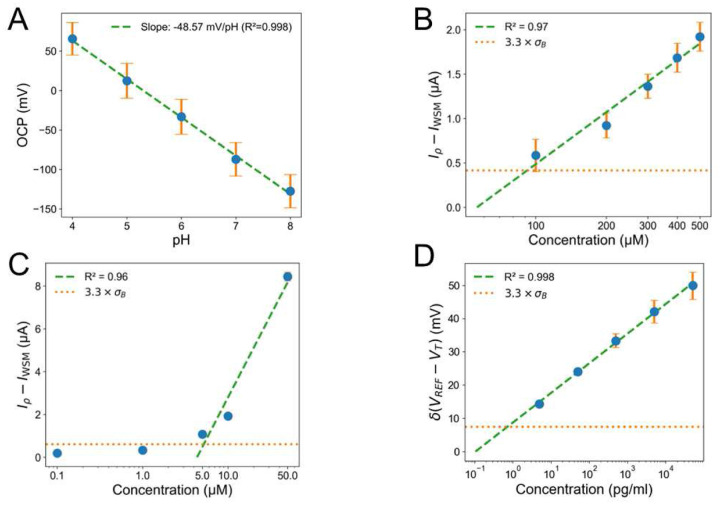
In-vitro detection of wound biomarkers in WSM. **(A)** Calibration plot of OCP versus pH (n = 3). Calibration curves obtained using DPV after baseline subtraction to detect **(B)** UA (n = 9) and **(C)** PCA (n = 9). **(D)** Response to recombinant human IL-6 calculated based on the baseline-subtracted gate voltage at *I_DS_*= 150 μA (n = 6). The error bars show the standard errors. The dashed line represents linear fitting, and the dotted line represents the standard deviation of the blank solution (*σ_B_*).

After our initial studies in PBS, the sensors were tested in wound-mimicking environments: a WSM and a thin layer of agar gel acting as a wound-like *in-vitro* model. Similar to the PBS tests, we systematically studied different reference electrode preparations in WSM (Ag paste with and without PVB modification) and also with and without HCl surface cleaning, for details see Supporting Information, Table S4 and Figure S10. Our results show that Ag paste without PVB and cleaned with HCl offers the most stable response which is consistent with the PBS results. The PANi/LIG-based pH sensor exhibited a sensitivity of $-48.57\;\frac{mV}{pH}$ (*R*^2^ = 0.99, *n* = 3) within a pH range of 4 − 8 in WSM (Figure 2 (A)). A decreased sensitivity compared to the response in McIlvaine buffer is attributed to the complexity and high ionic strength of WSM, which contains agar (5%), bovine plasma (25%), and lysed horse blood (25%), as also reported previously.^73^ The high ionic strength compresses the activity coefficient of H^+^, leading to a decrease in the change of the electromotive force per pH unit and producing a slope that is below the ideal Nernstian value.^74^ In addition, Figure S11 shows the OCP variation with stepwise changes in pH from 4 to 8 and cycled back to pH 4.

We also evaluated the UA/PCA electrochemical sensors in spiked WSM. Figure 2 (B) shows the sensitivity to UA which is decreased from $19.16\;\frac{\mu A}{dec}$ in 1× PBS to $1.94\;\frac{\mu A}{dec}$ in WSM. Besides, the LOD slightly increases from 64.14 *μM* in 1× PBS to 92.8 *μM* in WSM. Although the sensor sensitivity to UA is significantly lower in WSM, its response to PCA (sensitivity = $7.70\;\frac{\mu A}{dec}$, and LOD = 5.21 μ*M*) is comparable to the performance in 1× PBS in Figure 2 (C). LIG-based EGFET sensors also show a similar performance in WSM, with *S*_*v*_ and LOD of $8.91\;\frac{mV}{dec}$ and $0.72\frac{pg}{ml}$, respectively, with a good linearity (*R*^2^ = 0.99) over IL-6 concentrations of $5\;\frac{pg}{ml}$ to $50\;\frac{ng}{ml}$ (Figure 2 (D)). Example transfer characteristic of EGEFT sensors in WSM is shown in Figure S7 (B). Similar to 1× PBS, the IL-6 EGFET sensors show < 0.1 mV hysteresis in WSM (Figure S8 (B)).

Following sensor evaluation in liquid media as a model for wound exudate, we assessed their performance with a thin agar gel layer applied over the electrodes, serving as an *in-vitro* model of the wound environment. We deposited 10 μ*l* of biomarker-spiked WSM on an agar slab (thickness of *t* = 2 *mm*, diameter of *d* = 6 *mm*), and flipped and placed it on the sensor – with the deposited sample in direct contact with the electrodes. To study the effect of diffusion of biomarkers on agar, we tested different incubation times (*T*_*inc*_), from 1 min to 30 min. According to sensor signals shown in Figure S12, *T*_inc_ = 15 min was chosen.

**Figure 3.**
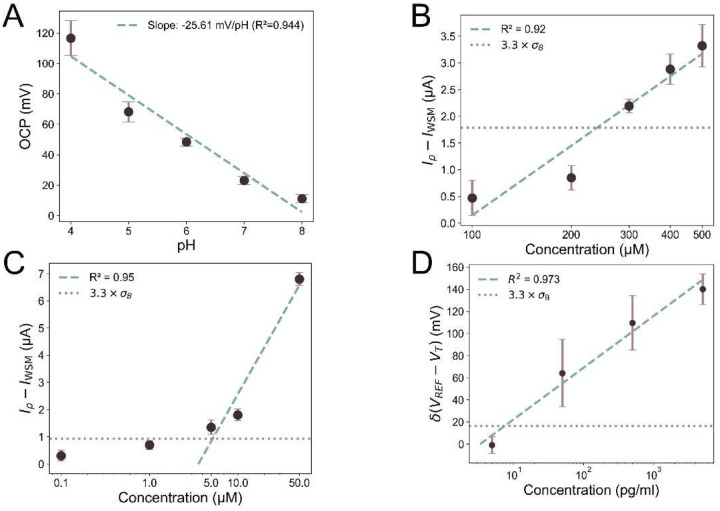
In-vitro detection of wound biomarkers on agar gel in WSM. **(A)** Calibration plot of OCP versus pH (n = 3) with flipped, biomarker-spiked WSM on agar. Calibration curves obtained using DPV after baseline subtraction to detect **(B)** UA (n = 9) and **(C)** PCA (n = 9). **(D)** Response to recombinant human IL-6 calculated based on the baseline-subtracted gate voltage at *I_DS_*= 150 μA (n = 9). The error bars show the standard errors. The dashed line represents linear fitting, and the dotted line represents the standard deviation of the blank solution (*σ_B_*).

Figure 3 (A) illustrates the performance of the pH sensors on agar, with a sensitivity of $-25.61\;\frac{mV}{pH}$ over pH of 4 to 8. This reduction in sensitivity is likely a result of the agar layer hindering ion transport, which in turn slows down the protonation and deprotonation processes of the PANi-based LIG electrode. Additionally, the electrochemical sensor exhibits a sensitivity and LOD of $4.33\;\frac{\mu A}{dec}$ and 239.58 μ*M* for UA, and $5.74\;\frac{\mu A}{dec}$ and 5.21 *μM* for PCA, respectively (Figure 3 (B) and (C)). Notably, sensor response to PCA does not change significantly in the presence of agar gel, in contrast to UA, particularly in terms of LOD. This discrepancy may be attributed to the larger molecular size of UA and its slower diffusion through the gel, limiting its accessibility to the electrode surface.

Given the complexity of agar gel compared to liquid samples (PBS or WSM), for detection of IL-6, we first evaluated the performance of EGFET with 1-pass vs. 2-pass LIG.^65^ While not significantly different in WSM, the sensitivity and LOD of 2-pass LIG is better compared to 1-pass LIG in agar tests (Figure S13), which is believed to be due to improved analyte transport, higher crystallinity, reduced surface roughness, and balanced defect density, electrical conductivity, and mechanical stability. Using 2-pass LIG, EGFET devices achieved sensitivity and LOD of $46.82\;\frac{mV}{dec}$ and $7.54\;\frac{pg}{ml}$, respectively, in agar tests from $5\;\frac{pg}{ml}$ to $5\;\frac{ng}{ml}$, Figure 3 (D).

### 2.5 Multimodal Sensor System for Multiplexed Detection of Wound Biomarkers

**Figure 4.**
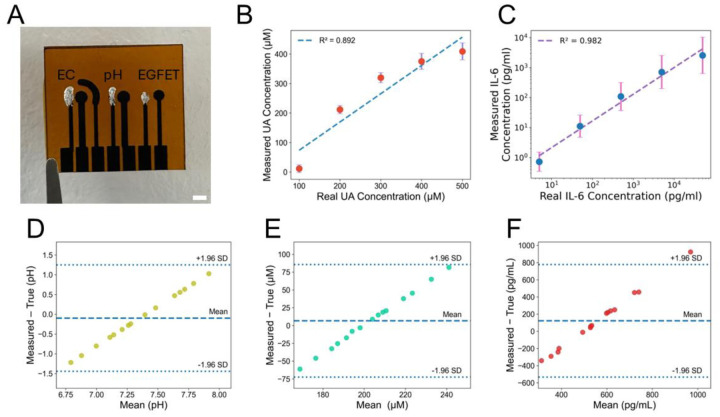
Multiplexed and simultaneous detection of wound biomarkers using an integrated multimodal sensor system, confirming minimal interference across different modalities. **(A)** Optical image of the integrated sensor system for multiplexed detection of pH, UA, and IL-6. Scale bar: 2 mm. **(B)** Baseline-subtracted DPV signals in response to varying concentrations of UA in the presence of $50\;\frac{pg}{ml}$ of IL-6 versus the same in the absence of IL-6 (n = 9). **(C)** Baseline-subtracted gate voltage at I_DS_ = 150 μA for different IL-6 concentrations in the presence of 200 μ*M* UA versus the same in the absence of UA (n = 9). **(D)** Bland–Altman plot comparing the quantified pH values with the pH measured in the presence of UA (200 μ*M*) and IL-6 $\left(500\;\frac{pg}{ml}\right)$ at pH = 7.4, n = 15. **(E)** Bland–Altman plot comparing the quantified UA concentration with the UA measured in the presence of IL-6 $\left(500\;\frac{pg}{ml}\right)$ at pH = 7.4, n = 15. **(F)** Bland–Altman plot comparing the quantified IL-6 concentration with the IL-6 measured in the presence of UA (200 μ*M*), at pH = 7.4, n = 15. The error bars show the standard errors. These experiments are performed in 1× PBS.

With each sensing modality being separately validated in various media, we then moved to assess the performance of the integrated sensor system (shown in Figure 4 (A)) for simultaneous detection of biomarkers. We first evaluated the individual performance of the electrochemical and EGFET sensors using separate biomarker solutions and verified minimal cross-interference between the sensing modalities. Figure S14 (A) demonstrates a clear, concentration-dependent DPV response for UA (in the absence of IL-6), whereas Figure S14 (B) confirms no observable DPV current response across different concentrations of IL-6 (in the absence of UA). Similarly, Figure S14 (C) verifies that the EGFET sensors do not respond to UA in the absence of IL-6, as expected. These initial control experiments confirmed that the two detection methods (electrochemical and EGFET) operate independently. Building on these results, Figure 4 (B) demonstrates that the electrochemical sensor responses to varying UA concentrations, both in the presence of a constant IL-6 concentration $\left(50\;\frac{pg}{ml}\right)$ and without IL-6, closely follow each other with an *R*^2^ of 0.89 in 1× PBS. Similarly, Figure 4 (C) illustrates the EGFET sensor response to varying concentrations of IL-6 in 1× PBS, both with and without UA (200 μ*M*), confirming stable and linear performance (*R*^2^ = 0.98). Thus, these experiments collectively validate the platform’s capability for simultaneous and interference-free detection of both electroactive and non-electroactive wound biomarkers.

We further validated our integrated sensor performance by detecting a single concentration of UA (200 μ*M*) and IL-6 $\left(500\;\frac{pg}{ml}\right)$ in 1× PBS (pH = 7.4). Bland–Altman plots shown in Figures 4 (D–F) indicate lower than ±25% error variation. This confirms the potential of the integrated sensor to detect multiple wound biomarkers with a user-friendly and affordable design.

**Figure 5.**
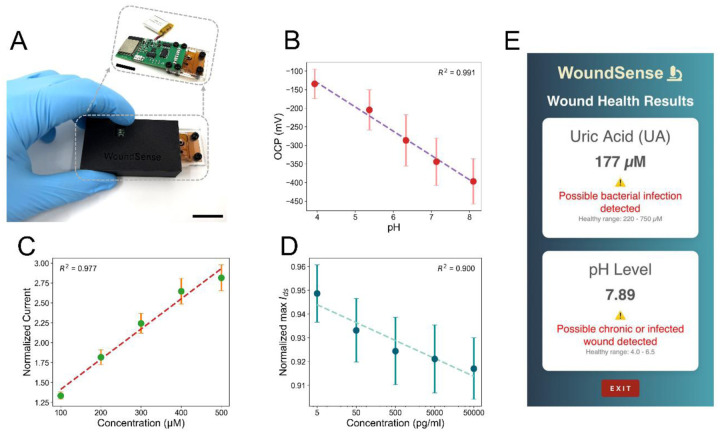
Wireless multimodal sensor system for simultaneous detection of wound biomarkers. **(A)** Optical image of the developed system. The scale bar is 2 *cm*.**(B)** Calibration plot of OCP versus pH (n = 5) in McIlvaine buffer. **(C)** Normalized UA peak current with baseline (1× PBS) calibration curves obtained using DPV (n = 7). **(D)** Normalized maximum I_*DS*_ responses to recombinant human IL-6, expressed as the ratio to the baseline maximum in 1× PBS (n = 4). **(E)** The results from the WoundSense app, estimating UA to be 177 μM and pH of 7.89, corresponding to a reference concentration of UA = 200 μM at pH 7.4. The error bars show the standard errors.

To demonstrate the potential of our integrated multimodal sensor system for point-of-care and remote monitoring, we developed a portable, wireless platform that seamlessly integrates pH, electrochemical, and EGFET-based sensors as depicted in Figure 5 (A). The integrated multimodal system is connected to the custom-designed wireless printed circuit board (PCB) module for real-time data collection (more information regarding PCB board design is provided in Methods: Design of the PCB for Portable and Wireless Multimodal Reading and Figure S15). Figure S15 (A) and (B) show the pin connections and PCB board design in top and back views of the wireless multimodal analyzer. To confirm the performance of our custom-designed wireless analyzer, we initially measured UA, pH, and IL-6 individually in 1× PBS. Figure 5 (B–D) demonstrates the successful performance of our portable multimodal system in detecting pH (4 − 8), UA (100 μ*M* − 500 μ*M*), and IL-6 $(5\;\frac{pg}{ml}-50\frac{ng}{ml}).$ To further demonstrate the system’s potential as a portable, stand-alone diagnostic device, we conducted wireless measurements using a custom-designed PCB interfaced with the biosensor and an accompanying mobile application (Figure S16). Specifically, we evaluated UA at 200 μ*M* with pH 7.4 and compared it to 400 μ*M* at pH 6 and 500 μ*M* at pH 5. As shown in Figure 5 (E) and Video S1, the sensor measured UA at 177 μ*M* and pH at 7.89, with an error variation below ±25%. Figure S17 (A-B) and Video S2 and S3 show the performance in response to UA concentrations of 400 μ*M* at pH 6 and 500 μ*M* at pH 5, with the system measuring UA st 396 μ*M* at pH 5.88 and 476 μ*M* at pH 4.82, respectively.

## 3. Discussion

In this study, we developed a portable and wireless multimodal biosensing platform based on LIG capable of simultaneous detection of critical wound biomarkers—uric acid, Phenazine-1-Carboxylic Acid, interleukin-6, and pH. By integrating electrochemical sensing with an extended-gate field-effect transistor configuration, the sensor chip demonstrated high sensitivity, low detection limits, and robust performance across physiologically relevant conditions, including wound-simulating media and tissue-mimicking agar gels. The incorporation of a custom wireless analyzer enables real-time, multiplexed data acquisition, facilitating continuous and minimally invasive wound monitoring. This platform addresses key limitations of traditional wound diagnostic methods by offering rapid, multiplexed, and on-site biomarker detection without the need for bulky laboratory equipment or centralized facilities. The sensor system’s compatibility with flexible substrates and mask-free fabrication further supports scalable production and wearable applications. Overall, our LIG-based multimodal sensor chip shows great promise for advancing personalized wound care by enabling timely detection of infection and inflammation, monitoring healing progression, and guiding clinical interventions. Future work will focus on in vivo validation, expanding the biomarker panel, and integration into smart wound dressings to realize fully autonomous, continuous wound management systems.

## 4. Experimental Section:

### Materials and reagents:

Uric acid (U0881), 1-Pyrenebutyric acid (PBA, 257354), N, N-Dimethylformamide (DMF, D4551), bovine serum albumin (BSA, A437), MES hydrate (M2933), TWEEN^®^ 20 (P1379), aniline (132934), pyocyanin (P0046), potassium chloride (KCl, P9333) and polyvinyl butyral (PVB, P110010) were all obtained from Sigma-Aldrich. EDC (1-ethyl-3-(3-dimethylaminopropyl) carbodiimide hydrochloride) (22980), sodium hydrogen phosphate (7558–79-4), citric acid (77-92-9), and LB Broth (Lennox) (H26760.36) were purchased from Thermo Scientific. N-Hydroxysulfosuccinimide sodium salt (Sulfo-NHS, H1304) was purchased from TCI. Isopropyl alcohol (IPA) (67-63-0) was obtained from VWR International, LLC. Hydrochloric acid (AC124210025) was obtained from Thermo Fisher Scientific. Recombinant human IL-6 protein (ab259381), and anti-IL-6 antibody (ab233706) were purchased from Abcam. Horse Blood Lysed (IGHSWBLS) was obtained from Innovative Research. Sterile Bovine Plasma (GTX73201) was purchased from GeneTex. Dulbecco’s Phosphate Buffered Saline (DPBS) 1× was purchased from Corning. DuPont^™^ Kapton^®^ polyimide (PI) film with a thickness and width of 0.005″ and 12″, respectively, was obtained from American Durafilm. Glassy Ag/AgCl (3M NaCl) reference electrode (MF-2052) was purchased from BASI research products. Ultrapure deionized (DI) water (18.2 MΩ cm) was used in all of our experiments.

### LIG Fabrication:

Initially, the PI sheet is rinsed with IPA, dried with clean, dry air, and secured to the metal workbench of the commercial laser cutter (VLS2.30, Universal Laser Systems, Inc.) using tape along the edges. For optimal conductivity and structural integrity, the laser beam is precisely focused, and the parameters of laser power, speed, and resolution are set to 12.6%, 5.5%, and 1000 pixels per inch (ppi), respectively, in raster mode (see Figure S1 (A)). Our laser printer can achieve a maximum power and speed of 30 *W* and $762\;\frac{mm}{s}$

For 1-pass LIG, a single raster scan was performed using the above parameters to form the electrodes. For 2-pass LIG, the same laser settings were applied twice consecutively on the working and extended gate electrodes for electrochemical and EGFET configuration, respectively. In this study, 1-pass LIG was employed for the pH and electrochemical sensors targeting UA and PCA, as well as for EGFET devices tested in 1× PBS and wound-simulating media. 2-pass LIG was utilized specifically for the EGFET sensors evaluated on agar gel, following the rationale described in the Results and Discussion section. Unless specifically mentioned, we have used 1-pass LIG in developing different components of the sensors.

The pRE is created by applying silver-conductive epoxy adhesive (MG Chemicals 8331D), which is cured for 1 hour on a hot plate at 70°C. Subsequently, silicone (Ecoflex 5, Smooth-On, Inc.) is applied between the sensor area and the electrical contact pads to passivate the sensor.

### LIG Characterization:

To characterize the electrode surface morphology, quality, and structural properties of LIG, we conduct scanning electron microscopy (SEM) and Raman Spectroscopy, as illustrated in Figure S1 (A) and (B). In Figure S1 (B), the SEM images confirm the porous structure of LIG. Figure S1 (C) shows three main peaks of LIG at approximately 1345 *cm*^−1^, 1580 *cm*^−1^, and 2690 *cm*^−1^, which correspond to the D-band, G-band, and 2D-band, respectively, as revealed through Raman analysis.

### pH Sensor Fabrication:

The pH sensor was based on a pH-sensitive PANi film electropolymerized on the LIG electrode. Initially, the working electrode was electrochemically cleaned through 10 cycles of cyclic voltammetry (CV) at a scan rate of $100\;\frac{mV}{s}$ in 0.5 M HCl, spanning from −0.1 to 0.9 *V* (see Figure S2 (A)). Following this, a 100 *μl* solution consisting of 1 M HCl with 0.1 M aniline was subjected to 12 CV cycles from −0.2 to 1 *V* with a scan rate of $100\;\frac{mV}{s}$ to facilitate PANi electro-polymerization. After removing this solution, a fresh one was used for another 12 CV cycles (refer to Figure S2 (B)). Finally, the pH sensor was allowed to air dry overnight. In all CV experiments, glassy Ag/AgCl and LIG were used as RE and counter electrode (CE), respectively.

### Preparation of the Modified Reference Electrode for pH Sensor:

First, LIG was printed in the reference electrode area. A layer of Ag paste was then applied to the LIG surface and dried at 70 °C for one hour. Next, a solution of PVB/KCl/methanol was prepared by dissolving 86 mg of PVB and 300 mg of KCl in 1 mL of methanol. An aliquot of 9 μL of this solution was drop-cast onto the dried Ag paste. After the solvent evaporated and a gel-like layer formed, a 3 M KCl solution was applied to the electrode surface and allowed to stabilize for 24 hours. After incubation, the excess KCl solution was removed, and the electrode was thoroughly rinsed with deionized water to remove any precipitated salts. The electrode was then dried under gentle nitrogen gas. Lastly, the working electrode was printed and electropolymerized with PANi, and the entire device was passivated to finalize the fabrication process.

### Electrochemical Measurements:

MultiPalmsens (BASi Inc.) was used as commercial potentiostat for conducting CV and DPV measurements. PANi film electropolymerization on the LIG electrode utilized a three-electrode setup (working electrode (WE): LIG, counter electrode (CE): LIG, and reference electrode: glassy Ag/AgCl). Open-circuit potential (OCP) was measured using a two-electrode configuration (WE: LIG, and pRE), with pH adjustments made every 5 minutes. The DPV analysis of UA and PCA was conducted using a three-electrode arrangement (WE: LIG, CE: LIG, and pRE), scanning from −1 V to 1 V with a 5 mV step, at a scan rate of 10 mV/s, and involved three scans.

### EGFET Configuration and Gate Functionalization for IL-6 Detection:

The EGFET developed in this study consists of LIG (1-pass or 2-pass, depending on the application which is specified in the manuscript) as the sensing component and a commercial n-channel FET as the transducer, with the transistor gate connected externally to the LIG electrode serving as the EG. The sensing compartment of the EGFET features two LIG-based electrodes: the EG and the pRE. The measurements utilized two Keithley 6430 instruments to apply gate and drain voltages. A commercial MOSFET (CD4007UBE, Texas Instruments, DigyKey) served as the transducer, with the drain voltage fixed at 0.1 V. The gate voltage was swept from 0 to 2.5 V with step of 0.05 V to ensure operation in the linear regime. For EGFET devices measured in 1× PBS and wound-simulating media, 1-pass LIG was used as the extended gate, while 2-pass LIG was employed for agar-gel experiments to achieve improved performance, as described in the Results and Discussion.

To functionalize LIG as the extended gate (EG), first, 10 μ*L* of PBA in dimethylformamide (DMF; 5 *mM*) is drop-cast on LIG-EG and incubated for 2 hours in a humidity chamber (97% humidity). After 2 hours of incubation, the electrode is rinsed with DMF, then IPA, followed by deionized water, and finally dried with nitrogen gas. Next, 10 μ*L* of 0.4 *M* EDC/0.1 *M* Sulfo-NHS in 25 *mM* MES buffer is applied to the electrode and incubated for 35 minutes in a humidity chamber (97% humidity). To prepare this solution, 7.668 *mg* of EDC and 2.173 *mg* of Sulfo-NHS are dissolved in 100 μ*L* of MES. The electrode is then rinsed with MES buffer and dried using nitrogen gas. Following this, 10 μ*L* of Anti-IL-6 $\left(25\;\frac{\mu g}{ml}\right)$ is drop-cast on LIG-extended gate and incubated for 3 hours at 4°*C*. After incubation, the LIG-EG is rinsed with 1× PBS and dried with nitrogen gas. Subsequently, 10 μ*L* of BSA is drop-cast onto the electrode and incubated for 1.5 hours at ambient conditions. To prepare the BSA solution, 2.5 *mg* of BSA powder is dissolved in 2.5 *mL* of 1× PBS and 2.5 μ*L* of Tween, then stored at 4 °*C* for 15 − 30 minutes to fully dissolve. After the BSA incubation, LIG-EG is rinsed with 1× PBS and dried with nitrogen gas. Finally, different concentrations of recombinant human IL-6 protein (from $5\;\frac{pg}{ml}$ to $50\;\frac{n}{ml}$) are applied to the EG electrode, followed by electrical measurements. Before initiating measurements, the IL-6 protein solution is gently pipetted over the electrode for 30 seconds and incubated for 2.30 minutes three times.

### Design of the PCB for Portable and Wireless Multimodal Reading:

At the core of the PCB is an ESP32 microcontroller, selected for its built-in analog-to-digital and digital-to-analog converters, as well as its integrated Bluetooth and Wi-Fi communication capabilities. A CP2104 USB-to-serial interface connects the ESP32 to a PC via a micro-USB port, which also serves as the charging input for a 350 mAh Li-Po battery. Battery charging is managed by an MCP73831T charge controller, enabling portable operation for up to 6 hours; ideal for remote or field-based sensing tasks. Power regulation is handled by an AP2112–3.3V voltage regulator, ensuring stable 3.3V supply to all onboard components. The designed LIG sensor is integrated using spring-loaded gold contacts for reliable electrical connection. Extended-gate field-effect transistor sensor is measured using CD4007UBE MOSFETs due to their suitable electrical characteristics. The pH voltage signal is buffered using operational amplifiers (TLC2264). For voltammetry, transimpedance amplifier circuits convert the working electrode current output into a readable voltage. These signals are digitized by the ESP32’s ADCs and transmitted wirelessly via Bluetooth. All components used in the system are commercially available and sourced from DigiKey.

### Data Analysis:

Data processing was performed in Python using pandas (data handling), matplotlib (graphing), numpy (numeric processing), and scipy (regression, peak window search, interpolation). Baseline correction was applied using a fourth-degree polynomial with a custom iterative fitting approach, as well as third-degree polynomial fits implemented with the pybaselines package. Peak detection was performed using PeakUtils.

### Material Characterization Instruments and Setups:

Scanning electron microscopy (SEM) images were captured with an Apreo instrument from Thermo Fisher Scientific. Furthermore, Raman spectroscopy was conducted using a Horiba LabRam device based in Kyoto, Japan, which featured a 100x objective and an 1800 g/mm grating. A 532 nm laser was utilized at 5% of its maximum capacity of 110 mW. The Raman data were analyzed using LabSpec 6 software.

### Use of AI Tools in Manuscript Preparation:

During the preparation of this work, we utilized ChatGPT (driven by OpenAI’s GPT-4 language model; http://openai.com) for two primary reasons: (1) to aid in structuring the outline and enhancing the manuscript for grammatical precision and better readability, and (2) to assist in creating conceptual illustrations for Scheme 1 and Figure S1a based on our detailed descriptions. In every instance, the authors reviewed, revised, and approved the final outputs to ensure scientific accuracy and take complete responsibility for the content provided.

## Supplementary Material

Supporting Information is available from the Wiley Online Library or from the author.

Supplementary Files

This is a list of supplementary files associated with this preprint. Click to download.

- SIAsghariannpj2DMaterialsandApplicationsSensingwith2DMaterialscollection.docx
- VideoS1.mp4
- VideoS2.mp4
- VideoS3.mp4

## Data Availability

The data that support the findings of this study are available from the corresponding author upon reasonable request.
